# Affective Responses From Different Modalities of Resistance Exercise: Timing Matters!

**DOI:** 10.3389/fspor.2019.00005

**Published:** 2019-08-02

**Authors:** Daniel J. Cavarretta, Eric E. Hall, Walter R. Bixby

**Affiliations:** ^1^Department of Exercise Science, Elon University, Elon, NC, United States; ^2^Department of Health, Fitness, and Exercise Studies, Anne Arundel Community College, Arnold, MD, United States

**Keywords:** affective responses, resistance exercise, emotion, weightlifting, machines, free weights

## Abstract

Resistance exercise provides positive changes in affect that may increase adherence. Little is known about the temporal dynamics of affect or the relationship between training modality and affect. This experiment investigated the temporal dynamics of affect during resistance exercise and compared the affective responses from machine and free weight exercises. Twenty-eight novice lifters (21 females) completed 2 workouts consisting of 4 machine or 4 free weight exercises for 3 sets of 9–11 repetitions at 80% 10 repetition-maximum. Feeling Scale was administered at baseline, during, 5- and 30-min post. During the workout, Feeling Scale was administered during the seventh repetition of the second set and after completion of the third set to provide an intra- and inter-set affective measurement. A Repeated Measures General Linear Model revealed a significant effect for time (*p* < 0.001) with affect more positive for all time points, 5- and 30-min following exercise compared to baseline levels (*p*'s < 0.001). Additionally, affect was more positive at 5- compared to 30-min post (*p* = 0.015) and higher for the inter-set measurement compared to the intra-set measurement (*p* = 0.001). The results suggest that affective valence becomes more positive during and following resistance exercise. This preliminary evidence suggests affective rebounding may occur after cessation of the set. Lastly, there appears to be no differences in the affective responses from machine and free weight exercises among novice lifters although this finding may be confounded by other factors such as differences in muscle group selection or total amount of volume performed.

## Introduction

Resistance exercise (RE) has long been accepted as a method to reduce the risk of cardiovascular disease, diabetes, and stroke and to enhance muscular size and strength (Westcott, 2012; Steele et al., 2017). This form of exercise has grown in popularity and has a great potential to have a positive impact on public health. In an effort to increase rates of participation in resistance training regimens, it may be important to focus on the affective responses experienced during exercise as research in aerobic exercise has found this to be linked to adherence (Williams et al., 2008; Rhodes and Kates, 2015). The affective responses from exercise are the feelings of pleasure and displeasure experienced during an exercise bout (Williams et al., 2008). The current body of evidence suggests that RE performed with moderate loads [50–70% one repetition-maximum (RM)] is perceived as pleasant both during and following exercise (Arent et al., 2005; Focht et al., 2015; Cavarretta et al., 2018).

Two areas in exercise psychology that warrant further investigation are the temporal dynamics of the affective experience of RE and the effect of training modality on affect (Cavarretta et al., 2018). Early research in exercise psychology measured affect or affective constructs (e.g., anxiety, mood) before and after aerobic exercise and concluded that “exercise makes people feel better” (Ekkekakis and Brand, 2019). Once researchers began measuring affect *during* aerobic exercise, conclusions became more elusive. While recent research has measured affect during resistance exercise (Focht et al., 2015; Greene and Petruzzello, 2015; Portugal et al., 2015), all previous experiments have done so after the completion of a set and not during a loaded muscular contraction. Investigating how affect changes throughout a set may have important implications for developing theories to explain the affective phenomena of RE. If affect is found to be different during a set of RE compared to after the cessation of the set, it will be important to question why this is. It is possible that affect is related to intramuscular pH, electromyographic amplitude, or velocity of movement. Understanding these relationships may lead to improved RE guidelines that will be accompanied by higher rates of adherence. For example, research in aerobic exercise has identified the ventilatory threshold as an important marking point of the transition to negative affect (Ekkekakis et al., 2011). Given that the ability to hold a conversation comfortably [e.g., Talk Test (Foster et al., 2008)] is related to the ventilatory threshold, practitioners can use this guideline to ensure that an exerciser is exercising below their ventilatory threshold. This maximizes the chances of having the exerciser experience a positive affective response during aerobic exercise.

An additional purpose of this experiment is to examine if there are differences in resistance training modality on the affective responses. Research in aerobic exercise has shown participants to report a more positive affective response to their preferred mode of exercise (Parfitt and Gledhill, 2004; Bixby and Lochbaum, 2008). Machine (MA) and free weight (FW) exercises are two popular modalities of RE. FW exercises tend to elicit greater muscle activation and hormonal response than MA exercises (Escamilla et al., 2001; Rossi et al., 2016). According to the Dual-Mode Theory, these differences in interoceptive cues may lead to differences in the affective response (Ekkekakis, 2003). If one of these modes of RE produces greater feelings of pleasure during exercise, it may be best to focus on that mode when making recommendations for RE.

## Methods

### Participants

The participants of this study were eligible if they were physically healthy and had not engaged in more than one session of RE per week for the last year. Thirty-three participants began the study, but five participants dropped out due to schedule difficulties (*n* = 4) or injury (*n* = 1; not due to this protocol). The 28 participants who completed the study were predominantly female (75%) and Caucasian (86%). Participant characteristics are presented in Table 1. Participants were recruited for this study from a private southeastern university. Prior to participating in this study, all participants read and signed an informed consent approved by the university's Institutional Review Board.

**Table 1:** Physical characteristics of participants (Means ± SD).

**Variable**	**Males (*n* = 7)**	**Females (*n* = 21)**
Age (years)	22.6 ± 4.6	23.4 ± 8.6
Height (cm)	177.1 ± 4.7	162.8 ± 5.1
Weight (kg)	79.4 ± 13.5	64.6 ± 15.9
BMI (kg/m^2^)	24.9 ± 4.9	24.4 ± 5.7

### Measures

Affective valence was measured with the Feeling Scale (FS; Hardy and Rejeski, 1989). FS is an 11-point scale ranging from −5 (“Very Bad”) to +5 (“Very Good”). FS has been used extensively to assess affect during exercise and is correlated with other measures of affective valence (Van Landuyt et al., 2000).

### Procedure

All participants completed two exercise conditions (MA and FW) presented in a randomized, counterbalanced fashion. Each condition consisted of two workouts for a total of four sessions. The first session for each condition determined the 10 RM for each exercise following guidelines by Haff and Triplett (2016). The 10 RM test was chosen as it is more appropriate for untrained participants than a 1 RM test and is in coherence with the goal repetitions of the study (Haff and Triplett, 2016). The first attempt was performed at ~50% of the participant's estimated 10 RM. The load was gradually increased until the participant could not complete 10 repetitions with proper form. All participants reached their 10 RM in no more than 5 attempts and were given 2–4 min of rest in between each attempt. The second session in each condition consisted of completing 3 sets of 9–11 repetitions at 80% 10 RM for each exercise with 90 s of rest in between each set. A range of repetitions was provided to account for variations in when participants would reach exhaustion. The researcher instructed the participant to perform at least 9 repetitions and provided no encouragement for the participant to continue or end the set. FS was measured before, during, and at 5- and 30-min post-exercise in a quiet room adjacent to the exercise facility. Additionally, there was both an intra- and inter-set measurement of affect during the exercise bout. The intra-set measurement was assessed after the completion of the seventh repetition of the second set by having the participant pause briefly and estimate their affective valence using FS. The seventh repetition was chosen as it represents a point in the repetition scheme where fatigue is beginning to accumulate and the lifter is near, but not at, momentary concentric failure. The inter-set affective measurement was assessed after the completion of the third set for each exercise.

All exercise sessions began with a 10-min warm-up on a recumbent bicycle with no resistance. The participant received at least 72 h of rest after completing the 10 RM and at least 48 h of rest after completing the workout at 80% of their 10 RM. A longer recovery period after completing the 10 RM was elected as there appears to still be decrements in performance 48 h after training to failure (Morán-Navarro et al., 2017). All participants were instructed on the proper technique for each exercise and received feedback for improper form during exercise; however, no verbal encouragement was given during the experimental sessions as to not influence the affective responses during the session.

All MA exercises were performed on Cybex VR3 equipment (Medway, MA, USA) and were performed in the order: leg press, row, chest press, and leg curl. All FW exercises were performed with standard barbells except for the goblet squat where the participant was instructed to vertically hold a dumbbell. If the barbell was too heavy for the exercise, a lighter ETS fixed straight barbell (York, PA, USA) was provided. The FW exercises were performed in the order: goblet squat, row, chest press, and stiff-leg deadlift. The goblet squat was taken until parallel squat depth and was selected as it may be easier for beginner weightlifters to learn than the barbell squat. These exercises were chosen to target all of the major muscle groups and the order of the exercises was chosen to alternate between pushing and pulling exercises.

### Statistical Analysis

All statistical analyses were conducted using IBM SPSS, Version 25.0. A Repeated Measures General Linear Model (RM GLM) was conducted to examine if there were differences in the number of repetitions completed between the two conditions. A RM GLM was conducted to determine if there were differences between pre- and post-RE in affect between conditions as measured by the FS. A RM GLM was performed to determine if there were differences in FS during the exercise session and between the conditions; this analysis included an examination of both intra-set and inter-set ratings of FS. Preliminary analyses did not find any main effects for gender; therefore, this was not included in the analyses presented.

## Results

### Volume

A 2 (conditions: MA vs. FW) × 4 (exercises: legs, rows, press, hamstrings) RM GLM for volume showed a significant main effect for condition, Wilks λ = 0.76, *F*_(1, 27)_ = 8.73, *p* = 0.006, but not for exercise or condition by exercise interaction. The effect for Condition was due to participants performing more volume of work in the MA condition compared to the FW condition. Table 2 displays the average number of repetitions performed for each set for each individual exercise.

**Table 2:** Average number of repetitions performed per set for each exercise (Means ± SD).

**Leg press**	**Machine row**	**Chest press**	**Leg curl**
10.12 ± 0.63	9.96 ± 0.72	9.94 ± 0.72	9.89 ± 0.71
**Goblet squat**	**Barbell row**	**Bench press**	**Stiff-leg deadlift**
9.70 ± 0.71	9.83 ± 0.79	9.71 ± 0.77	9.68 ± 0.78

### Affect

**Figure 1:**
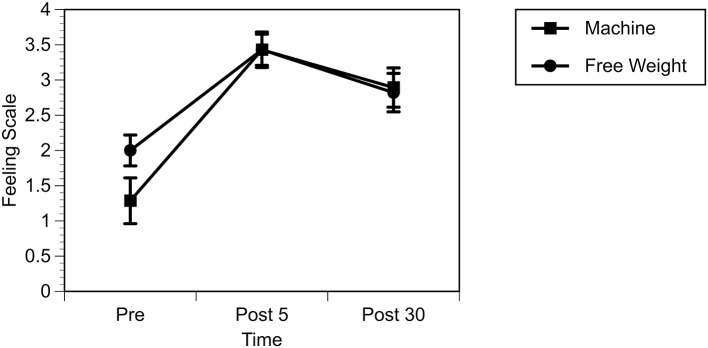
Feeling Scale responses over time and by condition.

A 2 (conditions: MA vs. FW) × 3 (time points: pre, post 5, and post 30) RM GLM for FS showed a significant main effect for Time, Wilks λ = 0.50, *F*_(2, 26)_ = 26.28, *p* < 0.001, but there was no condition or condition by time interaction. Fisher's LSD indicated that FS was increased from pre-exercise levels at post 5 following (*p* < 0.001), and post 30 (*p* < 0.001). FS was also higher at post 5 compared to post 30 (*p* = 0.015). See Figure 1 for a graphical depiction of these results.

**Figure 2:**
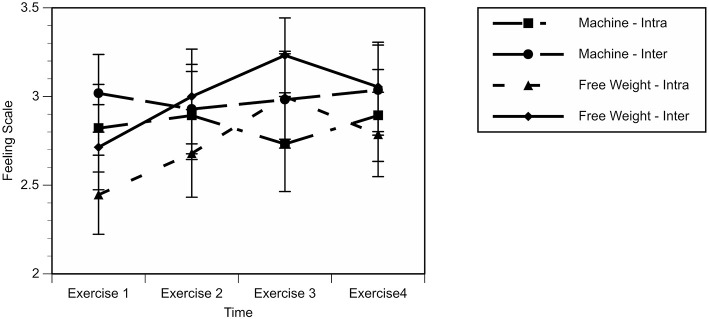
Feeling Scale (FS) during exercise session. Exercise one is the leg press and goblet squat; exercise two is machine row and barbell row; exercise three is chest press and bench press; and, exercise four is leg curl and stiff-leg deadlift.

A 2 (conditions: MA vs. FW) × 2 (time points: intra-set vs. inter-set) × 4 (exercises: legs, rows, press, hamstrings) RM GLM for FS showed a significant effect for Time, Wilks λ = 0.68, *F*_(1, 27)_ = 12.6, *p* = 0.001. No other main effect or interaction was found to be significant. The effect for Time was due to FS being significantly higher inter-set as opposed to intra-set. See Figure 2 for a graphical depiction of these results.

## Discussion

The results of this study suggest that RE provides improvements in affect up until at least 30-min post with affect peaking at 5-min post. Preliminary evidence suggests that affect may change throughout the course of a set as affect was less positive for the intra-set measurement compared to the inter-set measurement. One possible explanation for this finding is that the researcher caused a disturbance when they made the participant pause and respond to FS. Another explanation is that “affective rebounding” occurred. Affective rebounding is a phenomenon noticed in aerobic exercise where more than 95% of participants experience a positive affective response after the cessation of exercise (Ekkekakis et al., 2011). To the authors knowledge, this is the first experiment to show that affect experienced throughout the course of a set of RE may be different than affect experienced after the cessation of the set. Future experiments should investigate assessing affect at different time points throughout a repetition scheme and explore other measurements of affect, such as near-infrared spectroscopy (cf. Ekkekakis, 2009 for a review).

An additional finding of this experiment is that there was a similar positive affective response from performing both MA and FW exercises. Previously, Carraro et al. (2018) found a more positive affective response and higher ratings of enjoyment after completing a workout with FW as oppose to MA. This study recruited males with at least 2 years of experience with resistance training which differs from the present study which recruited 75% females with limited resistance training experience. As has been discovered with aerobic exercise, the affective responses from RE among experienced and novice lifters may be different (Bixby and Lochbaum, 2006; Hallgren et al., 2010). Additionally, volume was not equated in the present study and participants voluntarily performed a greater relative volume^1^ for the MA condition compared to the FW condition (71.84 vs. 70.10). Although this difference is small, it was statistically significant and this difference could partly explain the results of the study.

This study is not without limitation. While the exercises in the MA and FW conditions were matched based on the primary movers of each lift, it is nearly impossible to exactly match each exercise stimulus. FW exercises require greater balance and stability as there tends to be more degrees of freedom for a given movement. Specifically, with the exception of the bench press, all FW exercises involved the trunk musculature which was largely absent in the respected MA exercise. Movement patterns, absolute load, and volume did differ between each of the four exercises which may have influenced the changes in affect. Therefore, the direct comparison of MA to FW exercises is limited. Furthermore, affect was only assessed up until 30-min after completing the workout. The affective benefits of RE have been observed up to 60-min post-workout (Miller et al., 2009); and based on research on the anxiolytic and mood changing effects of RE, the benefits may extend up to 180-min or longer (Focht and Koltyn, 1999).

It has previously been recommended that novice lifters should begin resistance training with MA based movements before experimenting with FW based movements (Cavarretta et al., 2018). Based on data from this present study and Carraro et al. (2018), this recommendation does not appear to be substantiated and novice lifters may want to experiment with both modalities of RE. This recommendation is supported by other lines of research showing increasing the variety of exercise equipment to increase exercise participation and enjoyment (Juvancic-Heltzel et al., 2013). Additionally, a 6-week intervention showed no differences in exercise adherence for following a program consisting of MA or FW exercises (Faries and Lutz, 2016). Individual differences should be taken into consideration and practitioners should consider the lifter's preference of modality of exercise when designing a resistance training regimen. Furthermore, future research should consider including intra-set measurements of affect to better assess the affective experience while performing loaded muscular contractions.

## Data Availability

The datasets generated for this study are available on request to the corresponding author.

## Ethics Statement

Elon University 17-266—Comparison of the Effects of Machines and Free Weights on Affect and Anxiety.

## Author Contributions

DC collected data and prepared the manuscript. EH conducted the statistical analysis and wrote the results of the experiment. EH and WB provided feedback on the manuscript. All authors collaborated on the experimental design.

### Conflict of Interest Statement

The authors declare that the research was conducted in the absence of any commercial or financial relationships that could be construed as a potential conflict of interest.

## Footnotes

^1^Calculated as the sum of sets ^*^ average repetitions ^*^ 0.6 for each exercise.
